# ComX-Induced Exoproteases Degrade ComX in *Bacillus subtilis* PS-216

**DOI:** 10.3389/fmicb.2018.00105

**Published:** 2018-02-01

**Authors:** Mihael Spacapan, Tjaša Danevčič, Ines Mandic-Mulec

**Affiliations:** Chair of Microbiology, Department of Food Science and Technology, Biotechnical Faculty, University of Ljubljana, Ljubljana, Slovenia

**Keywords:** cell signaling, protease, quorum quenching, biofilms, degradative enzymes, quorum sensing, auto inducing signal, pellicles

## Abstract

Gram-positive bacteria use peptides as auto-inducing (AI) signals to regulate the production of extracellular enzymes (e.g., proteases). ComX is an AI peptide, mostly known for its role in the regulation of bacterial competence and surfactant production in *Bacillus subtilis*. These two traits are regulated accordingly to the bacterial population size, thus classifying ComX as a quorum sensing signal. ComX also indirectly regulates exoprotease production through the intermediate transcriptional regulator DegQ. We here use this peptide-based AI system (the ComQXPA system) as a model to address exoprotease regulation by ComX in biofilms. We also investigate the potential of ComX regulated proteases to degrade the ComX AI peptide. Results indicate that ComX indeed induces the expression of *aprE*, the gene for the major serine protease subtilisin, and stimulates overall exoprotease production in biofilms of *B. subtilis* PS-216 and several other *B. subtilis* soil isolates. We also provide evidence that these exoproteases can degrade ComX. The ComX biological activity decay is reduced in the spent media of floating biofilms with low proteolytic activity found in the *comP* and *degQ* mutants. ComX biological activity decay can be restored by the addition of subtilisin to such media. In contrast, inhibition of metalloproteases by EDTA reduces ComX biological activity decay. This suggests that both serine and metalloproteases, which are induced by ComX, are ultimately capable of degrading this signaling peptide. This work brings novel information on regulation of exoproteases in *B. subtilis* floating biofilms and reveals that these proteolytic enzymes degrade the AI signaling peptide ComX, which is also a major determinant of their expression in biofilms.

## Introduction

Auto-inducing (AI) signaling provides the means to integrate information on cell density, mass transfer and other environmental parameters by sensing secreted signaling molecules (Redfield, 2002; Bassler and Losick, 2006; Hense and Schuster, 2015). AI is especially relevant in biofilms, where cell density is very high (Nadell et al., 2008; Hense et al., 2012; Estrela and Brown, 2013). Signaling molecules bind to specific receptors, which then induce transcription and consequently the synthesis of beneficial and secreted products termed public goods (e.g., proteases) (Brown and Taddei, 2007; Diggle et al., 2007; Hense and Schuster, 2015; Schuster et al., 2017). The AI regulation of extracellular proteases (exoproteases) has been most extensively studied in Gram-negative bacteria, especially *Pseudomonas aeruginosa* (Brint and Ohman, 1995; Redfield, 2002; Hense et al., 2007; Hense and Schuster, 2015). These bacteria usually use lactonases for self-degradation of AI signals, acyl homo-serine lactones (Fekete et al., 2010; Terwagne et al., 2013).

In Gram-positive bacteria AI systems employ peptide pheromones as signaling molecules (Kleerebezem et al., 1997), which induce many adaptive processes at critical cell concentrations including the production of exoprotease in *Staphylococcus aureus* (Tegmark et al., 1998; Boles and Horswill, 2008); or in *Bacillus subtilis* (Msadek et al., 1991; Stanley and Lazazzera, 2005). In *B. subtilis*, the *comQXPA* gene cluster encodes the major peptide based AI system (Weinrauch et al., 1990), which is wide spread in the phylum Firmicutes (Dogsa et al., 2014). Existing studies focus on the regulatory role of ComX in the expression of the *srf* operon, responsible for the synthesis of surfactin (lipopetide antibiotic) and on the development of genetic competence for transformation (Nakano et al., 1991; D’Souza et al., 1994; Lazazzera et al., 1997; Ansaldi et al., 2002; Stefanic and Mandic-Mulec, 2009; Oslizlo et al., 2014, 2015; Stefanic et al., 2015; Aleti et al., 2016; Pollak et al., 2016). The observations that the former traits are regulated mostly in a cell density dependent manner (Ansaldi et al., 2002; Oslizlo et al., 2015) made the ComQXPA system widely known as the quorum sensing (QS) system of *B. subtilis*. In the ComQXPA system, the activity of the histidine kinase response regulator pair, ComP – ComA, is modulated by a signaling peptide, ComX (Magnuson et al., 1994; Ansaldi et al., 2002; Okada et al., 2005). ComX is modified by the isoprenyl transferase ComQ (Tortosa et al., 2001; Ansaldi et al., 2002; Schneider et al., 2002; Okada et al., 2005). Extracellular accumulation of the modified ComX leads to phosphorylation of ComA and subsequent induction of the ComA regulon (Ogura et al., 2001; Comella and Grossman, 2005). The *degQ* gene is also part of this regulon (Msadek et al., 1991; Stanley and Lazazzera, 2005). DegQ enhances phosphorylation of the response regulator DegU (Kobayashi, 2007) by DegS (Dahl et al., 1992; Jers et al., 2011). The level of phosphorylated DegU is a key information needed for proper expression of the DegU regulon (Murray et al., 2009). High DegU-P positively regulates production of extracellular enzymes including exoproteases (Verhamme et al., 2007; Veening et al., 2008). The *aprE* gene encodes the major serine exoprotease of *B. subtilis* that is under direct DegU-P control (Kawamura and Doi, 1984; Veening et al., 2008). AprE together with the metalloprotease NprE accounts for 95% of all extracellular proteolytic activity in *B. subtilis* (Kawamura and Doi, 1984), which are also under negative control of several repressors (Barbieri et al., 2016). *B. subtilis* encodes four other minor exoproteases that are either metalloproteases or serine type proteases (Wu et al., 1991). Dependence of DegQ synthesis on ComA (Msadek et al., 1991) strongly suggests that ComX indirectly controls *aprE* transcription. Additionally, *aprE* is known to be expressed in biofilms (Marlow et al., 2014) and therefore it is of interest to study the link between the ComQXPA system and proteases in a biofilm setting, which has not been attempted yet to our knowledge.

Overall, we still lack a deeper understanding of the importance of the ComQXPA system in exoprotease production in *B. subtilis*. Therefore, the first part of this study will be concerned in establishing that ComX is indeed crucial for the production of exoproteases. Furthermore, since ComX is a signaling peptide, it should be susceptible to degradation in highly proteolytic environments. Therefore, we investigate this question in the second part of our study, by testing *in vitro* degradation of ComX by *Bacillus* exoproteases.

With the latter points in mind we present the two main hypotheses:

(1) Exoprotease production in *B. subtilis* populations with a non-functioning ComQXPA system will be severely diminished, because ComX plays a major role in the induction of exoprotease production.
(2) ComX will be degraded by the induced exoproteases, because as a peptide it should be subject to degradation in highly proteolytic environments.

The results section is organized in two parts, in the first part we present evidence supporting the first hypothesis, and in the second part we present evidence supporting the second hypothesis.

## Materials and Methods

### Bacterial Strains, Growth Media, and Growth Conditions

Bacterial strains used in this study are listed in **Table 1**. Overnight cultures were incubated at 37°C and shaken at 200 rpm in LB medium with the appropriate antibiotics. To express heterologous ComX, a 2% (v/v) of overnight culture of *Escherichia coli* ED367 grown in LB medium supplemented with 100 μg/ml ampicillin was inoculated into the fresh M9 minimal medium (Ansaldi et al., 2002). Cells were grown to OD_650_ of ∼0.7 a.u. at 37°C and 200 rpm. Then IPTG was added in final concentration 0.4 mM. Cells were incubated further for 4 h at 200 rpm and 37°C and then centrifuged at 8,000 *g* for 10 min. M9 spent medium was sterilized through filters with 0.2 μm pores and stored at 4°C until use.

**Table 1:** Strains used in this study.

Strain name	Background	Genome description	Reference
***Bacillus subtilis* strains**			
PS-31		Undomesticated strain	Stefanic and Mandic-Mulec, 2009
PS-53		Undomesticated strain	Stefanic and Mandic-Mulec, 2009
PS-196		Undomesticated strain	Stefanic and Mandic-Mulec, 2009
PS-216		Undomesticated strain	Stefanic and Mandic-Mulec, 2009
PS-218		Undomesticated strain	Stefanic and Mandic-Mulec, 2009
BD2876	168	*his leu met srfA-lacZ* (*tet*) *comQ*::*kan*	Tortosa et al., 2001
BD2962	168	*his met srfA-lacZ* (*tet) amyE*::*xylR* P*xyl-comK* (*cat*) (*comQ*::pED345 *comX comP* replaced by genes from *B. mojavensis* RO-H-1)	Tortosa et al., 2001
BD3019	168	*his leu met srfA-lacZ* (*tet*) *amyE*::*xylR* P*xyl-comK* (*cat*) (*comQ*::pED375 *comX comP* replaced by genes from *B. subtilis* RS-D-)2	Ansaldi et al., 2002
BM1289	PS-31	*comQ*::pED345 (*spec*)	This work
BM1290	PS-53	*comQ*::pED345 (*spec*)	This work
BM1291	PS-196	*comQ*::pED375 (*spec*)	This work
BM1292	PS-218	*comQ*::pED375 (*spec*)	This work
BM1400	PS-216	*comQ*::*kan*	This work
BM1402	PS-216	*comP*::*cat*	Oslizlo et al., 2014
BM1127	PS-216	Δ*comQ* marker less	This work
BM1133	PS-216	*degQ*::*tet*	This work
BM1445	PS-216	Δ*comQ degQ*::*tet*	This work
BM1142	PS-216	P*aprE-gfp* (cm)	This work
BM1144	PS-216	*degQ*::*tet* P*aprE-gfp* (cm)	This work
O8G57	168	P*aprE-gfp* (cm)	Veening et al., 2008
BM1443	PS-216	Δ*comQ* P*aprE-gfp* (cm)	This work
BM1448	PS-216	Δ*comQ degQ*::*tet* P*aprE-gfp* (cm)	This work
DL722	3610	*amyE*::P*srfAA-yfp* (spec)	López et al., 2009
BM1456	PS-216	*comQ*::*kan amyE*::P*srfAA-yfp* (spec)	This work
BD7123	168	*degQ*::*tet* P*comGA-luc* (cm)	Miras and Dubnau, 2016
***Escherichia coli* strains**			
ED367	BL21 (DE3)	pET22(b) – *comQ comX* from *B. subtilis* 168 (amp)	Ansaldi et al., 2002
DE553		pMiniMAD2 *ori^BsTs^ amp mls*	Parashar et al., 2013

To prepare *B. subtilis* spores, the overnight culture [1% (v/v)] was inoculated into sporulation medium (SM) (Warriner and Waites, 1999). After 5 days of incubation, the culture was exposed to 80°C for 30 min and then centrifuged at 10,000 *g* for 10 min. The pellet was re-suspended and washed three times with physiological saline. Before freezing at -20°C 10% (v/v) glycerol was added to the spore suspension. Spores were enumerated using the MPN method (data not shown). A 1% (v/v) solution of triphenyltetrazolium chloride (TTC; BioLife, Italy) was sterilized through filters with 0.2 μm pores. 180 μl of the liquid LB medium with TTC [0.01% (v/v) final concentration] was dispensed in standard 96-well sterile microtiter plates. A 10-fold serial dilution of the spore suspension was prepared in microtiter plates with eight technical replicates for each 10-fold dilution down to 10^-11^. Microtiter plates were then incubated at 37°C overnight. Positive wells were identified by red color development due to the bacterial growth. Spore suspensions with MPN count in the 10^8^ MPN/mL range were used in the experiments.

To measure gene expression in floating biofilms (pellicles) a spore suspension 1% (v/v) was inoculated into the liquid MSgg medium. In some experiments the MSgg medium was supplemented with 20% (v/v) of the spent medium containing ComX, which was heterologously produced by *E. coli* ED367 (Ansaldi et al., 2002). The spent M9 medium of *E. coli* ED367 that was not induced by IPTG was used as a negative control.

To harvest pellicle spent media 1% (v/v) of *B. subtilis* spores were inoculated in 4 ml of MSgg medium (Branda et al., 2001), in some cases complemented with 20% (v/v) spent M9 minimal medium and incubated in sterile 12-well microtiter plates in static conditions at 37°C.

### Strain Construction

Construction of the mutant strains was performed by transformation of specific markers into competent *B. subtilis* strains grown in competence medium (CM) at 37°C (Albano et al., 1987). Antibiotic selections were carried out on LB agar plates at 37°C containing chloramphenicol (Cm) 5 μg/ml, kanamycin (Kan) 50 μg/ml, spectinomycin (Spec) 100 μg/ml, tetracycline (Tet) 10 μg/ml, erythromycin 0.5 μg/ml, and lincomycin 12.5 μg/ml (*mls*). The *comQ*::*spec* mutants were constructed by transforming the *B. subtilis* PS-31, PS-53 with the DNA isolated from the strain BD2962 (Tortosa et al., 2001) and by transforming PS-196 and PS-218 with the DNA isolated from the strain BD3019 (Ansaldi et al., 2002). The *comQ*::*kan* mutant (BM1400) was constructed by transforming the *B. subtilis* PS-216 with the DNA isolated from the strain BD2876 (Tortosa et al., 2001). The *degQ*::*tet* mutant was constructed by transforming the genomic DNA from the strain BD7123 (Miras and Dubnau, 2016) to appropriate PS-216 strains. Mutants with P*aprE-gfp* were constructed by transforming O8G57 genomic DNA (Veening et al., 2008) into appropriate PS-216 strains. When constructing the *amyE*::P*srfAA-yfp* mutants, the genomic DNA from the strain DL722 (López et al., 2009) was transformed into the strain BM1400. When transforming DNA into *comQ* mutants, the competence was achieved by the addition of exogenous ComX in the form of spent medium [5% (v/v) of the *E. coli* ED367 M9, which was grown in the presence of IPTG].

To construct the Δ*comQ* marker less deletion strain, the region upstream of the *comQ* gene was PCR amplified using the primer pair Up-F/Up-R (**Table 2**) and digested with EcoRI and BamHI. Also the region downstream of *comQ* gene was PCR amplified using the primer pair Down-F/Down-R (**Table 2**) and digested with BamHI and SalI. The two fragments were then simultaneously ligated into the EcoRI and SalI sites of pMiniMAD2 (Parashar et al., 2013), which carries a temperature-sensitive origin of replication and an erythromycin resistance cassette to generate pMiniMAD2-updowncomQ. The constructed plasmid was transformed into *B. subtilis* PS-216 at the restrictive temperature for plasmid replication (37°C) using 0.5 μg/ml erythromycin and 12.5 μg/ml lincomycin (*mls*) as a selection. To evict the plasmid, the strain was harvested according to an established protocol (Patrick and Kearns, 2008). Chromosomal DNA from colonies that had excised the plasmid was isolated and screened by PCR using primers Up-F/Down-R to determine which isolates carried a deletion in *comQ* gene.

**Table 2:** Oligonucleotides used in this study.

Name	Sequence 5′–3′
Up-F	CCGGAATTCATGACAAAGCGAAAAGGCCAC
Up-R	CGCGGATCCCTCCTTCATTTTCTCCTTGATCCGGAC
Down-F	CGCGGATCCACAAGATGCAAGACCTAATTAACTAC
Down-R	ACGCGTCGACCCTATTTCTCCAAGGTATCTTTGTATA

### Estimation of the Proteolytic Activity Produced by a *B. subtilis* Colony

Skim milk powder 20% (w/v) was reconstituted with distilled water and autoclaved at 110°C. An agar solution 3% (w/v) was also prepared and autoclaved at 121°C. After autoclaving, the suspensions were carefully mixed in a 1:1 ratio. Finally 20 ml of the mixture was poured into 9 cm diameter Petri dishes, yielding 10% skim milk (w/v) and 1.5% (w/v) agar. For overnight cultures *B. subtilis* strains were incubated in LB medium supplemented with appropriate antibiotics at 37°C and 200 rpm. Cultures were then diluted 100-fold in physiological saline and a 5 μl droplet of diluted culture was surface spotted in the center of the skim milk agar plate. Photos of the proteolytic clearing zones were taken after 16 h of incubation at 37°C.

### Preparation of Casein-Gelatin Plates

To prepare casein gelatin agar plates 1% (w/v) casein sodium salt from bovine milk (Sigma–Aldrich, United States) and 1% (w/v) gelatin from porcine skin (Sigma–Aldrich, United States) were thoroughly dissolved in 0.02 M NaOH and the pH was equilibrated to 7 ± 0.2 as described by Montville (1983). Wherever, the proteolytic inhibition by ethylenediaminetetraacetic acid (EDTA) was tested, EDTA was added to the casein gelatin medium in 1 mM final concentration. Finally, 1.5% (w/v) of agar was added to the casein gelatin medium. The casein gelatin medium was autoclaved at 110°C. 40 ml of the medium was poured into Petri dishes with 9 cm diameter. Wells (6 mm diameter) were cut into the casein gelatin agar using an agar punch cutter.

### Determination of Proteolytic Activity in Spent Media of Floating Biofilms

Proteolytic activity was determined in *B. subtilis* spent medium at different time points. The spent medium below the biofilm was centrifuged for 5 min at 8000 *g* to remove remaining planktonic cells and sterilized through filters with 0.2 μm pores. Spent medium was diluted in 10 mM sodium acetate buffer with 5 mM calcium acetate (pH 7.5). If EDTA was added to experimental samples, spent medium was diluted in PBS buffer (10 mM sodium phosphate dibasic, 1 mM potassium phosphate monobasic, 137 mM NaCl, and 2.7 mM KCl pH 7). The diluted spent medium (100 μl) was dispensed into the wells punched into the casein gelatin agar. The plates were incubated for 16 h at 37°C when the proteolytic zones were measured. To estimate the proteolytic activity of spent media in U/ml of subtilisin, the proteolytic zone diameters of the spent media were compared to the proteolytic zone diameters obtained by different concentrations of commercial subtilisin (Sigma–Aldrich). One such subtilisin dose-response curve is shown in Supplementary Figure 1. The subtilisin concentration equivalents were displayed as units where 1 unit of subtilisin hydrolyzes casein to produce color equivalent to 1.0 μmol (181 μg) of tyrosine per minute at pH 7.5 at 37°C using Folin-Ciocalteu reagent. According to Sigma–Aldrich and to our in-house control of subtilisin proteolytic activity following the Sigma’s protease activity assay (Cupp-Enyard, 2008), 1 mg of subtilisin corresponds to roughly 13 units. Protease activity estimation was done routinely after storage of subtilisin for over 6 months at -20°C, to determine that the storage conditions did not lower the subtilisin standard proteolytic activity.

### Expression of P*aprE-gfp* during Formation of Floating Biofilm

Briefly, 200 μl aliquots of inoculated MSgg medium, sometimes supplemented with M9 spent medium were dispensed in a sterile 96-well black transparent bottom microtiter plate in four technical replicates. The lid was sealed with micropore tape and the space between wells was filled with sterile distilled water to minimize the effect of medium evaporation. The microtiter plate was incubated in the Cytation 3 imaging reader (BioTek, United States) at 37°C without shaking. Optical density at 650 nm and fluorescence intensity were measured in half hour intervals for up to 60 h. Fluorescence intensity of GFP (green fluorescent protein) was used to monitor P*aprE-gfp* expression with excitation at 480 nm and emission at 510 nm. The gain was set on 50. In a parallel experimental setup the same strains without the fluorescent marker were always cultured. To calculate the final expression the autofluorescence of unmarked strains was deducted from the fluorescence of the marked strains. Fluorescence intensity was normalized per OD_650_ of fluorescently labeled strains at each time point.

### The ComX Biological Activity Assay

The ComX biological activity was quantified in spent media of various *B. subtilis* strains harvested after 36 and 48 h of static growth, using biosensor strains: *B. subtilis* BM1456 and BM1400. We chose the 48 h time point because then all strains have comparable floating biofilm (pellicle) thickness, estimated by measuring OD_650_ (Supplementary Figure 2B). The BM1400 strain only served to determine background fluorescence, since it carries no fluorescent marker. Both biosensor strains do not produce their own ComX. The BM1456 biosenor responds to ComX in the spent medium by inducing the P*srfAA-yfp* reporter (Oslizlo et al., 2014). Since the induction of P*srfAA* is proportional to the quantity of ComX (Ansaldi et al., 2002) we used this assay to estimate the quantity of biologically active ComX in the spent media of various pellicles.

Both biosensor strains were grown in 5 ml of CM medium supplemented with 1% (v/v) of filtered spent media of selected strains for 6 h at 37°C with shaking (200 rpm). After 6 h of incubation 200 μl aliquots were dispensed in a sterile 96-well black transparent bottom microtiter plate. The P*srfAA*-*yfp* expression was quantified by YFP (yellow fluorescent protein) fluorescence intensity. YFP was excited at 510 nm and emissions were measured at 530 nm. The gain was set to 100. Each P*srfAA*-*yfp* reading was normalized to OD_650_. The normalized autofluorescence background of BM1400 grown in the same experimental conditions was subtracted. Additionally, we subtracted the P*srfAA*-*yfp* expression of cells exposed to Δ*comQ* spent medium (lacks ComX), to account for ComX independent YFP expression.

ComX biological activity was determined in the spent media immediately after harvest (T_0_) and after a 24 h (T_24_) incubation at 37°C. We reasoned that if exoproteases in the spent medium degrade ComX the ComX biological activity at T_0_ will be greater than at T_24_. To calculate the ComX biological activity decay we used the following equation:

$$\begin{array}{c} \mathrm{ComX}\mathrm{biological}\mathrm{activity}\mathrm{decay}[\%]=\;\frac{\mathrm{ComX}\mathrm{biological}\mathrm{activity}(T_{o})-\mathrm{ComX}\mathrm{biological}\mathrm{activity}(T_{24})}{\mathrm{ComX}\mathrm{biological}\mathrm{activity}(T_{o})}*100 \end{array}$$

The same approach was used to determine ComX biological activity decay in the spent media supplemented with subtilisin (0.1 mg/ml) or metalloprotease inhibitor EDTA (1 mM) after harvesting or to estimate the effect of commercially available subtilisin on ComX heterologously produced in *E. coli* ED367.

### HPLC Purification of Exoprotease Treated Spent Media

ComX was purified as described previously (Ansaldi et al., 2002; Oslizlo et al., 2014). Briefly, 5 ml of ED367 spent medium was acidified and separated with a C-18 reverse phase HPLC column. ComX presence was determined by measuring the absorbance at 214 nm, where it appears as two chromatographic peaks, both of which exhibit biological activity (Oslizlo et al., 2014). The medium was purified after adding subtilisin (0.1 mg/ml) and incubating it for 24 h at 37°C. To ensure heterologous ComX stability over time bovine serum albumin (BSA, 50 μg/ml) was added to the spent media prior to tests. The resulting chromatogram was compared to a control experiment, where subtilisin was not added. To control for the presence of other signals potentially produced by *E. coli* ED367, the spent medium of the non-induced *E. coli* was subjected to the same procedure.

### Statistical Analysis

All results were statistically analyzed using the non-parametric Mann–Whitney *U*-test. The threshold level for accepting significant differences was *p* = 0.05. All of the error bars show the standard error of mean (SEM).

## Results

### ComX Induces Exoproteases

#### The Regulation of P*aprE-gfp* Expression in Floating Biofilms Is ComQ and DegQ Dependent

Previous work suggested that ComA positively regulates expression of *degQ* that is needed for exoprotease production (Msadek et al., 1991). Furthermore, the DegU system was shown to be important for exoprotease activity during biofilm growth (Verhamme et al., 2007). Based on this knowledge we predicted that disruptions of *comQ*, required for ComX maturation, will cause a decrease in the transcriptional activity of the major protease gene in *B. subtilis* pellicles grown in MSgg medium.

**FIGURE 1:**
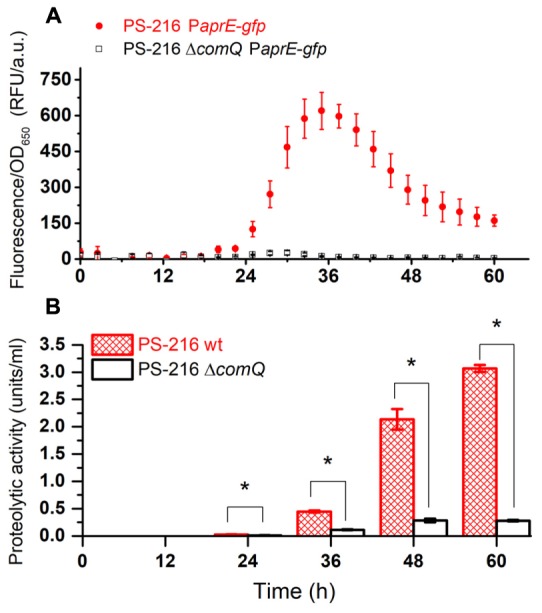
P*aprE-gfp* expression of floating biofilms grown in MSgg medium **(A)** and the proteolytic activity of harvested floating biofilm spent media **(B)**. **(A)** P*aprE-gfp* expression of floating biofilms grown in MSgg media was evaluated by measuring fluorescence. Measurements were performed every half an hour (only every fifth measured data point is shown for clarity). Averages and SEM (standard error of means) of four biological replicates are shown. **(B)** The proteolytic activity of floating biofilm spent media grown in MSgg media at different time points. Averages and SEM of three biological replicates are shown. A Mann–Whitney *U*-test was performed to determine statistical significance of discussed differences (^∗^*p* < 0.05).

In order to test this hypothesis, we monitored the population expression of P*aprE-gfp* of the wild type PS-216 strain and Δ*comQ* mutant grown as floating biofilms. P*aprE-gfp* expression is indeed higher in the wt strain compared to the Δ*comQ* mutant at all time points (**Figure 1A**). This is very similar to the P*aprE*-*gfp* expression in the *degQ*::*tet* mutant and Δ*comQ degQ*::*tet* double mutant. In all tested mutants P*aprE-gfp* expression is very low as compared to the expression in the wt strain (Supplementary Figure 2A). These results support the hypothesis 1 that ComQXPA AI system contributes to regulation of exoproteases. Also, expression of P*aprE-gfp* in the wt strain reaches maximal levels at 36 h of incubation (**Figure 1A** and Supplementary Figure 2A). This is the approximate time point where OD_650_ of wt strain and all mutant floating biofilms are comparable, despite their difference in surface morphology (Supplementary Figures 2B, 3). Nevertheless, P*aprE-gfp* expression remains significantly higher in the wt strain than in Δ*comQ* or *degQ*::*tet* mutants at early time points and even after 60 h of floating biofilm growth.

#### The Proteolytic Activity during Floating Biofilm Growth Is ComQ and DegQ Dependent

Then we measured the overall protease activity in the wt and Δ*comQ* mutant floating biofilm spent media (**Figure 1B**). Again, the exoprotease activity in the spent medium of the Δ*comQ* mutant is drastically lower than in the wt strain at all the measured time points. A significantly higher proteolytic activity of the wt strain than of the Δ*comQ* mutant spent medium is detectable already at 24 h and the difference increases with time. However, P*aprE-gfp* expression and proteolytic activity (**Figure 1**) do not correlate, especially after the 36 h incubation time point, but results further corroborate, that proteolytic activity in floating biofilm spent medium is ComQ dependent.

**FIGURE 2:**
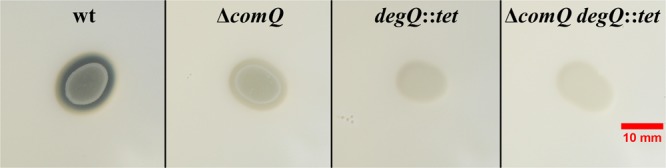
Proteolytic activity of *Bacillus subtilis* PS-216 wild type and Δ*comQ, degQ*::*tet*, and Δ*comQ degQ*::*tet* mutants on skim milk agar. Photos were taken on a dark blue background after 16 h of incubation at 37°C. Note the clearing zone around the wt colony, indicating the proteolysis of skim milk. Colonies of the mutant strains did not degrade skim milk as much and thus the clearing zones are less apparent.

This conclusion is further supported by experiments where we addressed the role of ComQ and DegQ in production of exoprotease during growth on skim milk agar. As predicted, wt colonies produced clearly visible proteolytic zones which were hardly visible in Δ*comQ, degQ::tet* and Δ*comQ degQ::tet* mutants (**Figure 2**). Also, different wt soil isolates of *B. subtilis* (PS-31, PS-53, PS-196, and PS-218) produce strong clearing zones around colonies grown on skim milk agar but lack them in Δ*comQ* isogenic mutants (Supplementary Figure 4). Therefore, the results support predictions made in the first hypothesis and reinforce the assumption of the importance of ComX in positive regulation of exoprotease production.

#### ComX Complements the Defect in P*aprE-gfp* Expression and Protease Activity in the *ΔcomQ* Mutant

**FIGURE 3:**
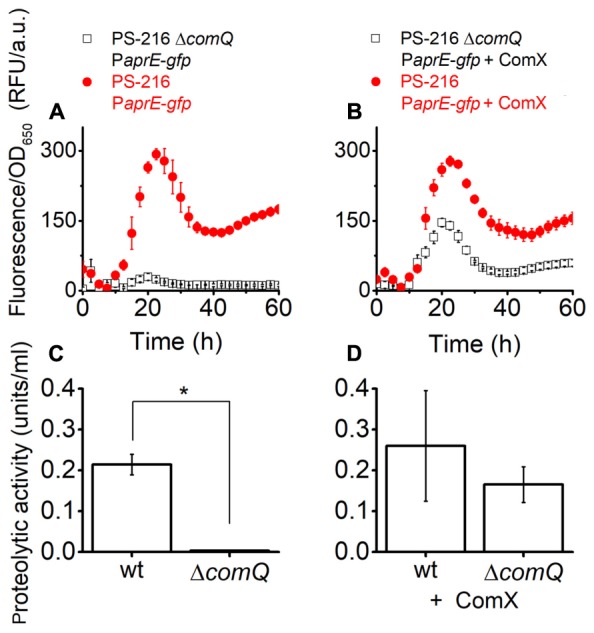
P*aprE-gfp* expression **(A,B)** and the proteolytic activity **(C,D)** of 24 h floating biofilm spent media of strains grown in MSgg medium complemented with spent M9 minimal medium. Cells were grown in MSgg medium with the addition of M9 *Escherichia coli* ED367 spent medium [20% (v/v)]. **(A,C)** Were complemented with spent M9 medium where ED367 heterologous expression of ComX was not induced with IPTG and therefore the spent medium lacked ComX. **(B,D)** Were complemented with M9 spent medium, where heterologous expression of ComX in ED367 was induced with IPTG, and therefore the spent medium contained heterologous ComX. Measurements on **(A,B)** were made every half an hour, only every fifth measured data point is shown for clarity. **(C,D)** Show the proteolytic activity of spent medium from floating biofilms of different PS-216 strains after 24 h growth in the same media as on **(A,B)** but in 12-well microtiter plates. A Mann–Whitney *U*-test was performed to determine statistical significance of discussed differences (^∗^*p* < 0.05). Averages and SEM (standard error of means) of three independent biological replicates are shown.

Next, we tested whether addition of ComX complements P*aprE*-*gfp* expression in the Δ*comQ* mutant. We added ComX in the form of a spent M9 minimal medium [20% (v/v)] of *E. coli* ED367, which heterologously produces ComX upon induction with IPTG. The M9 spent medium without IPTG induction was used as a control. Thus growth conditions for the complementation assay with M9 spent medium were slightly different as for results presented in **Figure 1** and Supplementary Figure 2. For example, OD_650_ measurements indicate that in MSgg + M9 medium floating biofilms start forming after 10 h (Supplementary Figure 5), while in MSgg medium this occurs after 20 h (Supplementary Figure 2B). This may explain different values for P*aprE*-*gfp* expression in MSgg + M9 medium (**Figures 3A,B**) compared to the ones presented in **Figure 1A**. Nevertheless, the addition of ComX complements P*aprE*-*gfp* expression and protease activity in the Δ*comQ* mutant (**Figures 3A,B**) and even restores the Δ*comQ* mutant floating biofilm morphology to the wt morphology at 24 h (Supplementary Figure 6). Although complementation was partial for P*aprE-gfp* expression, the spent media proteolytic activity at 24 h was also complemented by ComX (**Figures 3C,D**). In contrast and as expected, ComX does not complement P*aprE*-*gfp* expression in *degQ::tet* mutant and Δ*comQ degQ*::*tet* double mutant (Supplementary Figure 7), which is consistent with published results indicating that ComX works upstream of *degQ* (Msadek et al., 1991).

### Exoproteases Degrade ComX

#### Exoproteases in the Floating Biofilm Spent Media Contribute to the ComX Biological Activity Decay

ComX is a small peptide (Magnuson et al., 1994; Ansaldi et al., 2002), which implies that it might be susceptible to proteolytic degradation. If this is the case, we expect that the ComX biological activity decay will be high in exoprotease rich spent media of floating biofilms that produce exoproteases but low in the spent media of mutants that have low exoprotease activity (hypothesis 2).

**FIGURE 4:**
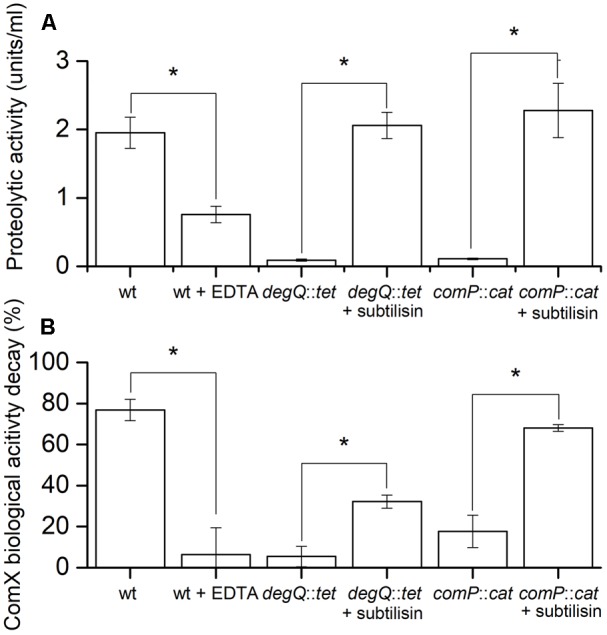
Comparisons of the proteolytic activity **(A)** and ComX biological activity decay **(B)** of different *B. subtilis* 48 h floating biofilm spent media. **(A)** Proteolytic activity of the spent medium from floating biofilms. Different *B. subtilis* PS-216 strains (wt, *degQ*:*tet, comP*::*cat*) were grown for 48 h at 37°C in MSgg medium. Where indicated, subtilisin or EDTA was added to spent media after harvest in order to increase or inhibit the proteolytic activity of the media, respectively. Averages and SEM of three independent biological replicates are shown. A Mann–Whitney *U*-test was performed to determine statistical significance of discussed measurements (^∗^*p* < 0.05). **(B)** The ComX biological activity decay of the same harvested floating biofilm spent medium as above using a signal deficient biosensor strain BM1456. Averages and SEM of three independent biological replicates are shown. A Mann–Whitney *U*-test was performed to determine statistical significance of discussed measurements (^∗^*p* < 0.05).

Floating biofilms of different strains were grown in 12-well microtiter plates and imaged at different time points. At 36 and 48 h the Δ*comQ* and *comP::cat* mutants form floating biofilms with a more structured surface than the wt strain and *degQ::tet* mutant, however, floating biofilm biomass appears similar (Supplementary Figure 3). Therefore, we chose these two time points (36 and 48 h) to measure the proteolytic activity and the ComX biological activity decay in the spent media of floating biofilms. Results show that at both time points proteolytic activity and ComX biological activity decay is high in spent media of the wt strain, but low in the spent media of the mutant strains (**Figure 4** and Supplementary Figure 8). This supports the second hypothesis. Furthermore, metalloprotease specific inhibitor EDTA (Ellaiah et al., 2002), added to the spent medium of the wt strain decreases the proteolytic activity (**Figure 4A**) and consequently the ComX biological activity decay (**Figure 4B**). This suggests that metaloproteases influence ComX stability. Finally, we tested whether subtilisin, which is the major serine protease produced by *B. subtilis* (Kawamura and Doi, 1984), can also degrade ComX. We added exogenous commercially available subtilisin to spent media harvested from *comP::cat* or *degQ::tet* mutants, which have otherwise very low proteolytic activities. Subtilisin increased proteolytic activity of mutant’s spent media to the levels of the wt strain (**Figure 4A**) and consequently also the ComX biological activity decay (**Figure 4B**). This supports the prediction that subtilisin, which expression is under ComX control, also modulates the ComX biological activity decay.

Summing up the results presented in **Figure 4** and Supplementary Figure 8, we conclude that the ComX biological activity decay in spent medium (i) coincides with the proteolytic activity of the media; (ii) is inhibited by metalloprotease inhibitor EDTA and (iii) is promoted by subtilisin. This speaks strongly in favor of our second hypothesis and implies that both, metalloproteases and subtilisin, degrade ComX.

#### The Protease Subtilisin Is Sufficient to Degrade ComX

**FIGURE 5:**
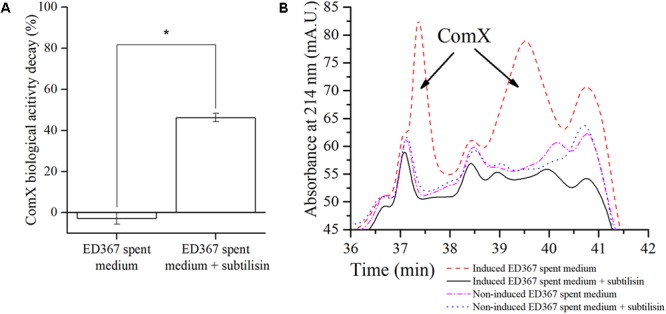
ED367 heterologous ComX biological activity decay **(A)** and the purification of heterologous ComX on HPLC **(B)**. **(A)** The ComX biological activity decay of the *E. coli* ED367 spent medium containing heterologously expressed ComX, using a signal deficient biosensor strain BM1456 with a P*srfAA-yfp* fluorescent reporter fusion. The spent medium was incubated for 24 h at 37°C with and without subtilisin. When adding subtilisin, the ComX biological activity decay increases. Averages and SEM of three independent biological replicates are shown. A Mann–Whitney *U*-test was performed to determine statistical significance of discussed measurements (^∗^*p* < 0.05). **(B)** Chromatograms of the *E. coli* ED367 M9 spent media that were incubated for 24 h at 37°C with and without subtilisin. After treating the medium with subtilisin, the two peaks containing ComX are no longer present in the medium that contained heterologously expressed ComX. As a control a parallel experiments were made where non-IPTG induced *E. coli* ED367 spent medium was incubated for 24 h at 37°C with and without subtilisin.

One might still argue, that exoproteases degrade ComX indirectly through some unknown intermediate factor, present in the native *B. subtilis* floating biofilm spent media. Therefore, to provide more direct experimental evidence of ComX degradation by exoproteases, we treated the *E. coli* ED367 M9 spent medium that contains heterologously expressed ComX with subtilisin (**Figure 5**). In the spent M9 minimal media from *E. coli*, such a confounding factor is less likely to be present. To test this, we incubated spent M9 media with heterologous ComX with or without subtilisin (0.1 mg/ml) for 24 h at 37°C. The results confirm that subtilisin increases the decay of the ComX biological activity as compared to the subtilisin untreated control (**Figure 5A**). Finally, one might argue that in all cases above no degradation of ComX occurs, only a modulation of the sensitivity of the ComX biosensor assay, since this is the only measure we used in order to quantify ComX so far. Therefore, in order to provide more direct proof of ComX degradation, we present the HPLC chromatograms of *E. coli* M9 spent media with heterologously expressed ComX, which were either treated with subtilisin or not (**Figure 5B**). We see that the chromatogram of the M9 minimal spent medium with ComX treated with subtilisin lacks the two distinct peaks (**Figure 5B**), which are indicative of ComX (Oslizlo et al., 2014). These two peaks are visible on the chromatograms of the M9 spent medium containing heterologous ComX induced by IPTG, but untreated with subtilisin. The chromatograms of additional negative controls, namely the ED367 spent media that were not induced by IPTG also lack the two ComX indicative peaks.

Overall these results speak strongly in favor of our second hypothesis and show that native exoproteases, which are according to hypothesis 1 induced by ComX, create a proteolytic environment that leads to the degradation of this AI signaling peptide.

## Discussion

**FIGURE 6:**
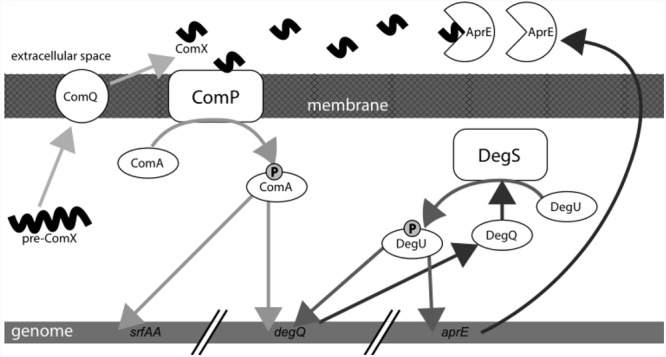
Schematic presentation of the quorum sensing (QS) ComQXPA system. ComX induces exoprotease production. Exoproteases ultimately degrade ComX in the extracellular milieu.

We here report that the ComQXPA AI system of *B. subtilis* positively controls the transcription of the subtilisin gene *aprE* and exoprotease production during biofilm growth at liquid–air interface or on agar surface. Moreover, we provide evidence, that exoproteases, which are induced by ComX in a DegQ dependent manner, can degrade ComX AI signaling peptide (**Figure 6**).

Many bacterial auto-inducing systems regulate the production of exoproteases (Diggle et al., 2007; Hense and Schuster, 2015; Schuster et al., 2017) and this regulation has mostly been studied in Gram-negative *Pseudomonas aeruginosa* (Brint and Ohman, 1995), but also in Gram-positive *Staphylococcus aureus* (Tegmark et al., 1998). We know less how AI systems regulate exoproteases in Gram-positive model organism, *B. subtilis*. The ComQXPA system has been implicated in expression of DegU regulon previously (Msadek et al., 1991; Stanley and Lazazzera, 2005) but the work was focused on planktonic cultures. We here investigate the role of ComX in exoprotease production during biofilm growth. Results confirm that ComX positively influences P*aprE*-*gfp* expression and exoproteases production in the model PS-216 strain and in a set of *B. subtilis* undomesticated wt strains, that were isolated from soil microscale (Stefanic and Mandic-Mulec, 2009) and differ in their physiology (Stefanic et al., 2012).

The Δ*comQ* mutant shows lower P*aprE*-*gfp* expression and proteolytic activity in floating biofilms (pellicles) at all time points compared to the wt strain. In this strain P*aprE*-*gfp* expression, but not proteolytic activity, starts decreasing in intensity after 36 h (**Figure 1A**). This drop is unexpected, because fluorescence measurements of promoter labeled strains usually indicate cumulative transcription (Miras and Dubnau, 2016). We also observe that the Δ*comQ* mutant forms pellicles at a faster rate than the wt strain up to 36 h (Supplementary Figure 2B). As the Δ*comQ* mutant is expected to have low levels of DegU-P leading to a more active primary metabolism (Tanaka et al., 2015) and faster growth. In addition, we note that after 36 h floating biofilms show indication of brown coloration (Supplementary Figure 3) indicative of sporulation (Koo et al., 2017). Changed physiological conditions may affect the stability of GFP. Nevertheless, this discrepancy does not compromise our main conclusion of the ComX AI system playing a significant positive role in expression/activity of exoproteases.

To our knowledge, this work also provides the first evidence that AI regulated exoproteases degrade the peptide auto-inducer ComX in *B. subtilis*. This is evident through experiments *in vitro* in which we show that ComX biological activity decay correlates with proteolytic activity of spent media. Both, serine proteases (e.g., subtilisin) and metalloproteases influence the ComX biological activity decay. Gram-negative bacteria use AHLs (acyl homoserine lactones) auto-inducing molecules for QS (Papenfort and Bassler, 2016). Quorum quenchers, like lactonases, degrade the signaling molecules in bacteria and often act as weapons in intra-species competition (Kalia, 2013; Hense and Schuster, 2015). Also in Gram-negative bacteria auto-degradation of AHLs is not well-understood. In *Brucella melitensis* a protein was identified, which has a potential for AHL degradation (Terwagne et al., 2013). Similarly, *Pseudomonas putida* accumulates AHL degradation by products during growth, indicating potential AHL degradation (Fekete et al., 2010). However, these findings, like ours for *B. subtilis*, show only the degradation of the AI molecule. The effect of such degradation on the microbial physiology is, to our knowledge, unknown.

Future experiments will show whether the self-directed AI signal degrading strategy is potentially a negative feedback regulatory loop of the ComQXPA auto-inducing response. The fact that the two most studied ComQXPA regulated traits, namely competence and surfactant production (Nakano et al., 1991; D’Souza et al., 1994; Lazazzera et al., 1997; Ansaldi et al., 2002; Stefanic and Mandic-Mulec, 2009; Oslizlo et al., 2014, 2015; Stefanic et al., 2015; Aleti et al., 2016; Pollak et al., 2016), are regulated in a cell density dependent manner (Ansaldi et al., 2002; Oslizlo et al., 2015) made the ComQXPA system widely known as the QS system of *B. subtilis*. However, if the proteolytic degradation of ComX has physiological consequences then this would imply that these traits may not be regulated in density dependent manner in conditions that support synthesis of exoproteases. This provides further support for the validity of the efficiency sensing theory (Hense et al., 2007; Hense and Schuster, 2015). This work describes two different types of regulation that involve the signaling peptide ComX: a positive genetic regulation and a negative biochemical regulation. The latter may represent a negative feedback loop, which could elegantly link the bacterial supply and demand for beneficial public goods. The bacterial population will increase public good supply via ComX. This will also increase proteolytic activity, which will decrease ComX and thus potentially further supply of public goods. If ComX is produced and degraded simultaneously, this may prevent cells to overinvest in protease production, which is more costly than the synthesis of a small peptide. Therefore, cells could potentially obtain information on the demand for exoproteases through ComX but this remains to be demonstrated.

To our knowledge this is the first report which links peptide-based AI system to production of exoproteases and subsequent self-degradation of the signaling molecules. We envision that protease dependent control of signaling peptides may be more wide spread among Gram-positive bacteria and hope that this work will initiate new research in this direction.

## Author Contributions

All authors (MS, TD, and IM-M) were involved in experimental design and the writing of the manuscript. MS and TD performed the experiments.

## Conflict of Interest Statement

The authors declare that the research was conducted in the absence of any commercial or financial relationships that could be construed as a potential conflict of interest.
