# Corrigendum: ComX-Induced Exoproteases Degrade ComX in *Bacillus subtilis* PS-216

**DOI:** 10.3389/fmicb.2018.01552

**Published:** 2018-07-03

**Authors:** Mihael Spacapan, Tjaša Danevčič, Ines Mandic-Mulec

**Affiliations:** Chair of Microbiology, Department of Food Science and Technology, Biotechnical Faculty, University of Ljubljana, Ljubljana, Slovenia

**Keywords:** cell signaling, protease, quorum quenching, biofilms, degradative enzymes, quorum sensing, auto inducing signal, pellicles

In the original article, there was an error.

Mutants in *amyE*::P_*aprE*_*-gfp* were constructed by transforming O8G57 genomic DNA (Veening et al., 2008) into appropriate PS-216 strains.

A correction has been made to Materials and methods, Strain Construction:

Mutants with P_*aprE*_*-gfp* were constructed by transforming O8G57 genomic DNA (Veening et al., 2008) into appropriate PS-216 strains.

In the original article, there was a mistake in Table 1, Figure 1, Figure 3, Figure S2, Figure S5, and Figure S7 as published. The genotypic trait P_*apre*_-*gfp* of the strains BM1142, BM1144, BM1443 and BM1448 was incorrectly stated as being *amyE*::P_*aprE*_*-gfp*.

The authors apologize for these errors and state that this does not change the scientific conclusions of the article in any way.

The original article has been updated.

## Conflict of interest statement

The authors declare that the research was conducted in the absence of any commercial or financial relationships that could be construed as a potential conflict of interest.
