# Supplementary material for: ComX-Induced Exoproteases Degrade ComX in Bacillus subtilis PS-216
Source: Front Microbiol. 2018 Feb 1;9:105. doi: 10.3389/fmicb.2018.00105 (PMC5799266; doi:10.3389/fmicb.2018.00105)
Supplement: Supplementary file 1 [file Presentation_1.pdf]

Supplementary Material

ComX-induced exoproteases degrade ComX in *Bacillus subtilis* PS-216

Mihael Spacapan[1], Tjaša Danevčič[1], Ines Mandic-Mulec[1*]

[1] Chair of Microbiology, Department of Food Science and Technology, University of Ljubljana Biotechnical Faculty, Ljubljana, Slovenia

*** Correspondence:**
Ines Mandic-Mulec
ines.mandicmulec@bf.uni-lj.si

1.1 Supplementary Figures

Subtilisin concentration (µg/ml)	6.3	4.2	2.8	1.9	1.2	0.8	0.5	0.4
Zone diameter (mm)	17.7	16.5	15.1	13.4	12.6	11.4	10.6	8.4

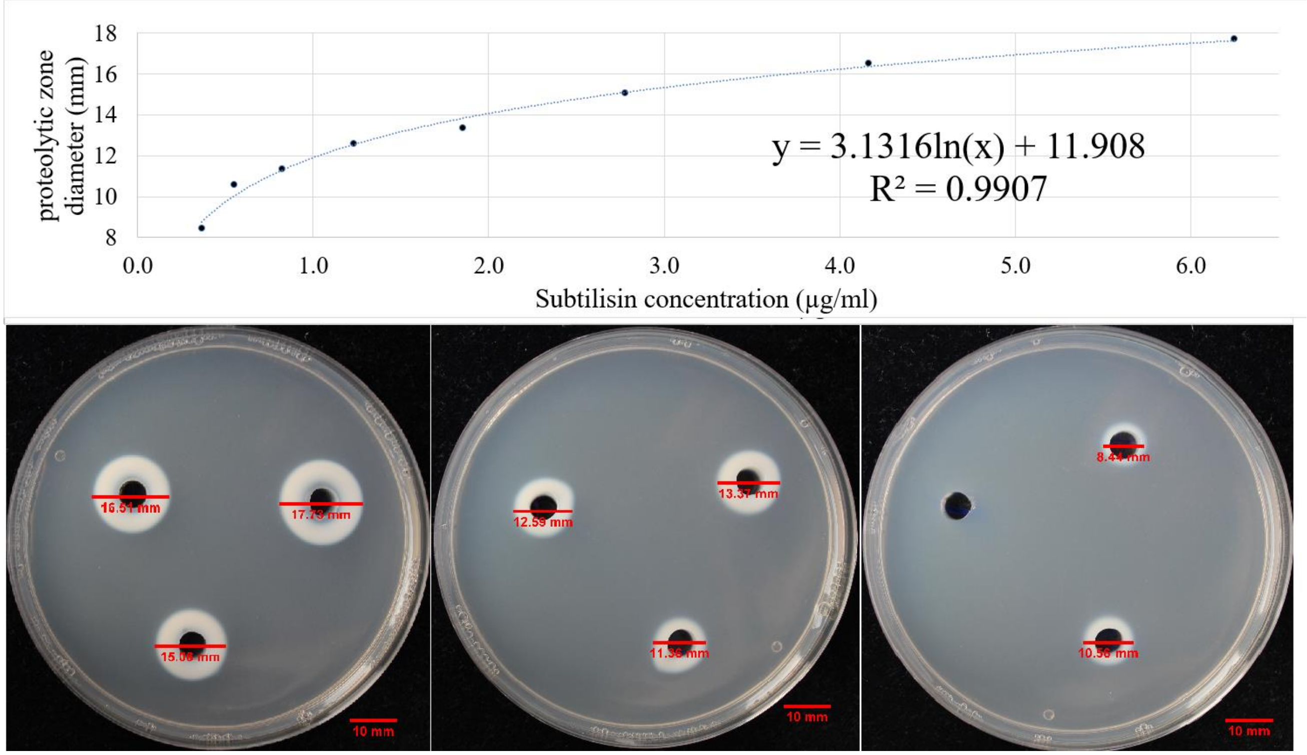

Supplementary Figure 1: A set of casein gelatin plates with the proteolytic zone diameters used for the standard dose response curve.

The measured diameters, with the FIJI is just ImageJ (FIJI) distribution (Schindelin et al., 2012) of ImageJ version 1.51d (Schneider et al., 2012), of each casein gelatin agar plates (bottom 3 photos) are summed up in a table on the top of the figure, which also indicates the concentration of subtilisin of the liquid dispensed in the agar wells. The dose-response curve is shown in the graph below the table. The natural logarithm fit equation and the R^2 value are also shown on the graph and were calculated using the Microsoft Excel software.

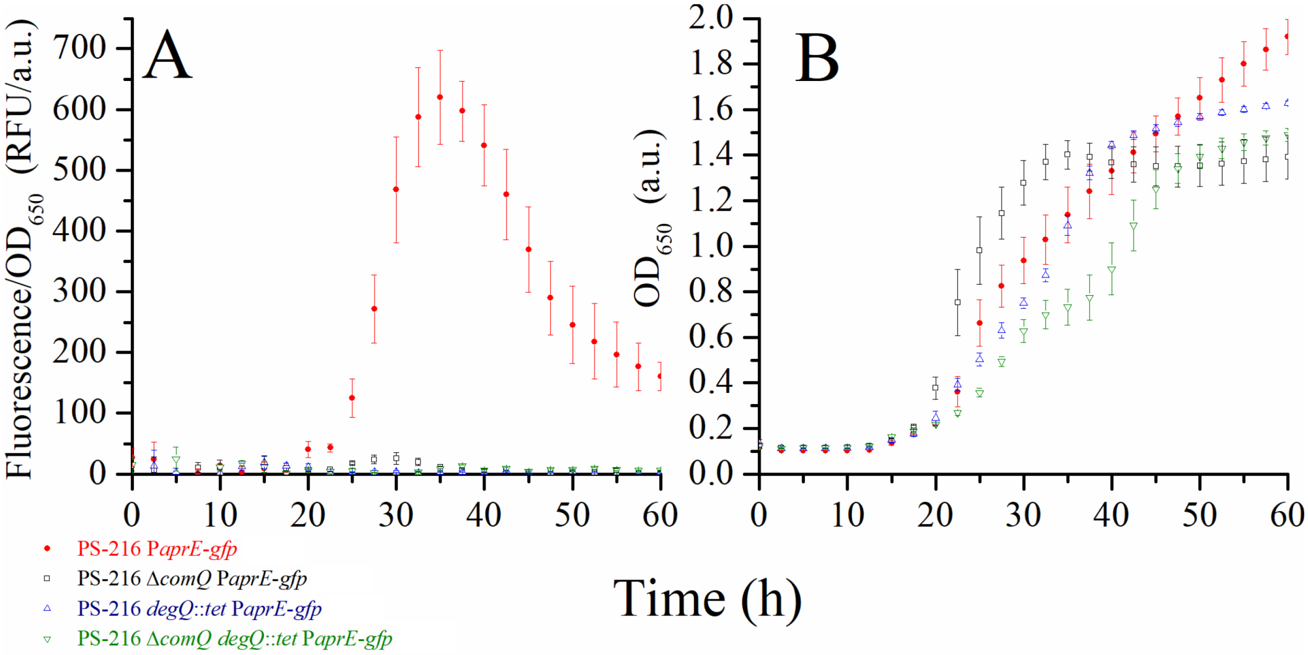

Supplementary Figure 2: P*aprE-gfp* expression (A) and optical density at 650 nm (B) of floating biofilms grown in MSgg medium

Cells were grown in MSgg medium as described in Materials and methods section 2.6 (Expression of P*aprE-gfp* during floating biofilm formation). The legend on the bottom left applies for both panels. Averages of 4 independent biological replicates with their standard error of means (SEM) are shown. Measurements were made every 30 minutes, but only every fifth data point is shown for clarity.

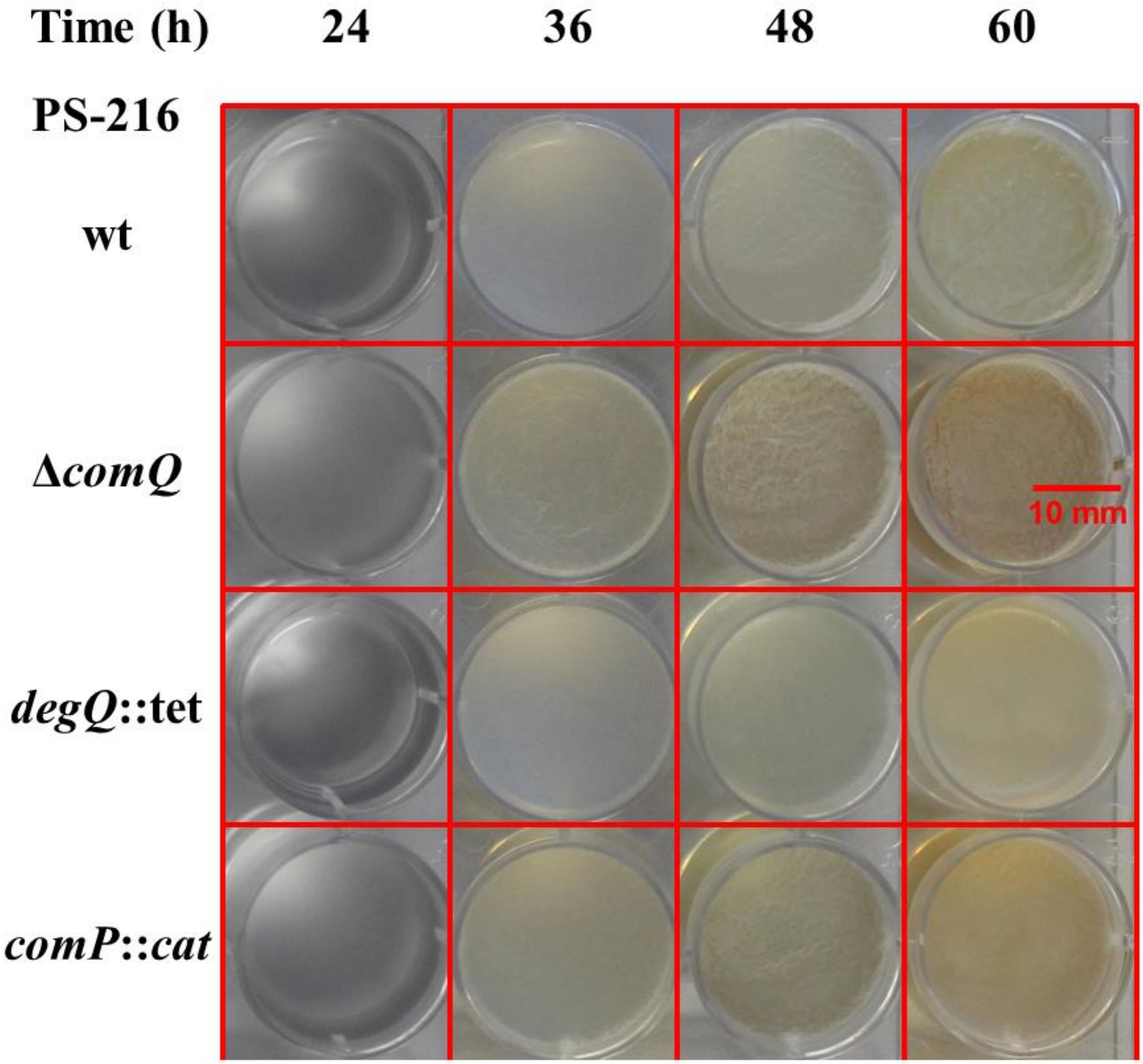

Supplementary figure 3: Floating biofilm morphologies of wt strain, Δ*comQ*, *degQ*::*tet* and *comP*::*cat* mutants after growth in MSgg medium in 12-well microtiter plates.

Floating biofilms of the PS-216 wt strain and of the Δ*comQ*, *degQ*::*tet* and *comP*::*cat* mutant strains grown in 12-well microtiter plates in MSgg medium, incubated in static conditions at 37 °C.

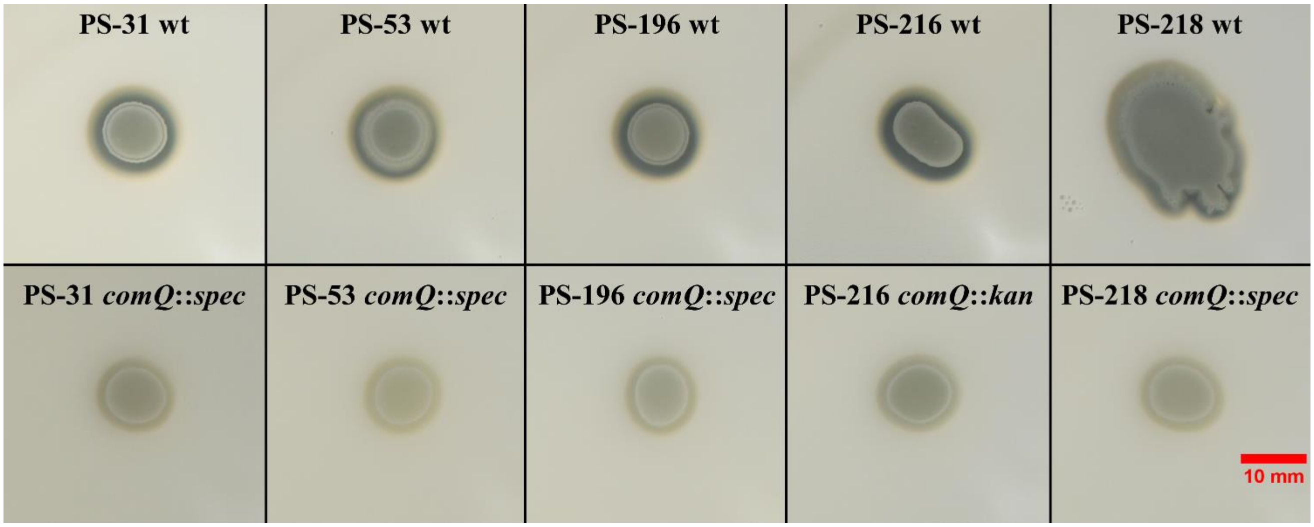

Supplementary figure 4: Proteolytic activity of *B. subtilis* soil isolates PS-31, PS-53, PS-196, PS-216 and PS-218 and their signal deficient counterparts grown as colonies on skim milk agar.

Proteolytic activity is visible as a clearing zone around wt colonies after 16 h of incubation at 37°C which is less apparent in the signal deficient *comQ* mutants. Photos were taken on a dark blue background.

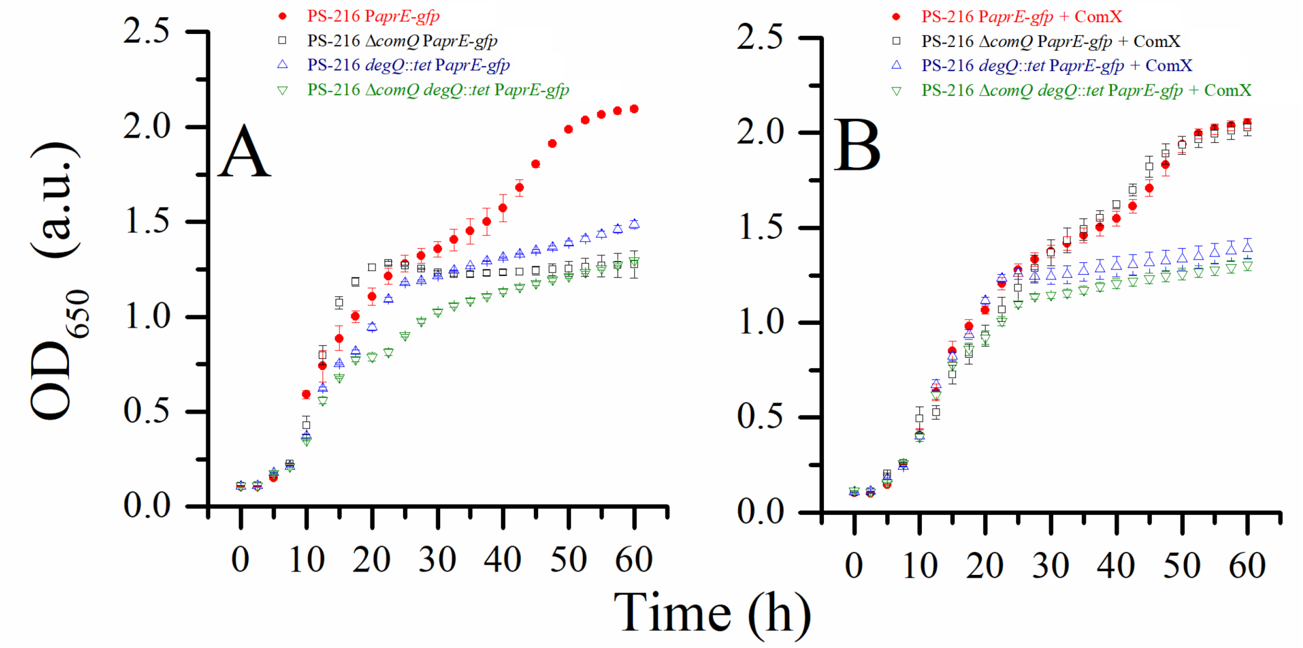

Supplementary Figure 5: Optical density at 650 nm of floating biofilms grown in MSgg medium complemented with 20% (v/v) spent M9 minimal medium with (A) or without (B) ComX.

The axis labels apply to both panels. In (A), MSgg medium was complemented with spent M9 medium where ED367 heterologous expression of ComX was not induced with IPTG and therefore the medium lacked ComX. In panel B, MSgg medium was complemented with spent M9 medium, where heterologous expression of ComX in ED367 was induced with IPTG, and therefore contained heterologous ComX. Averages of 3 independent biological replicates with their standard error are shown. Measurements were made every 30 minutes, but only every fifth data point is shown for clarity.

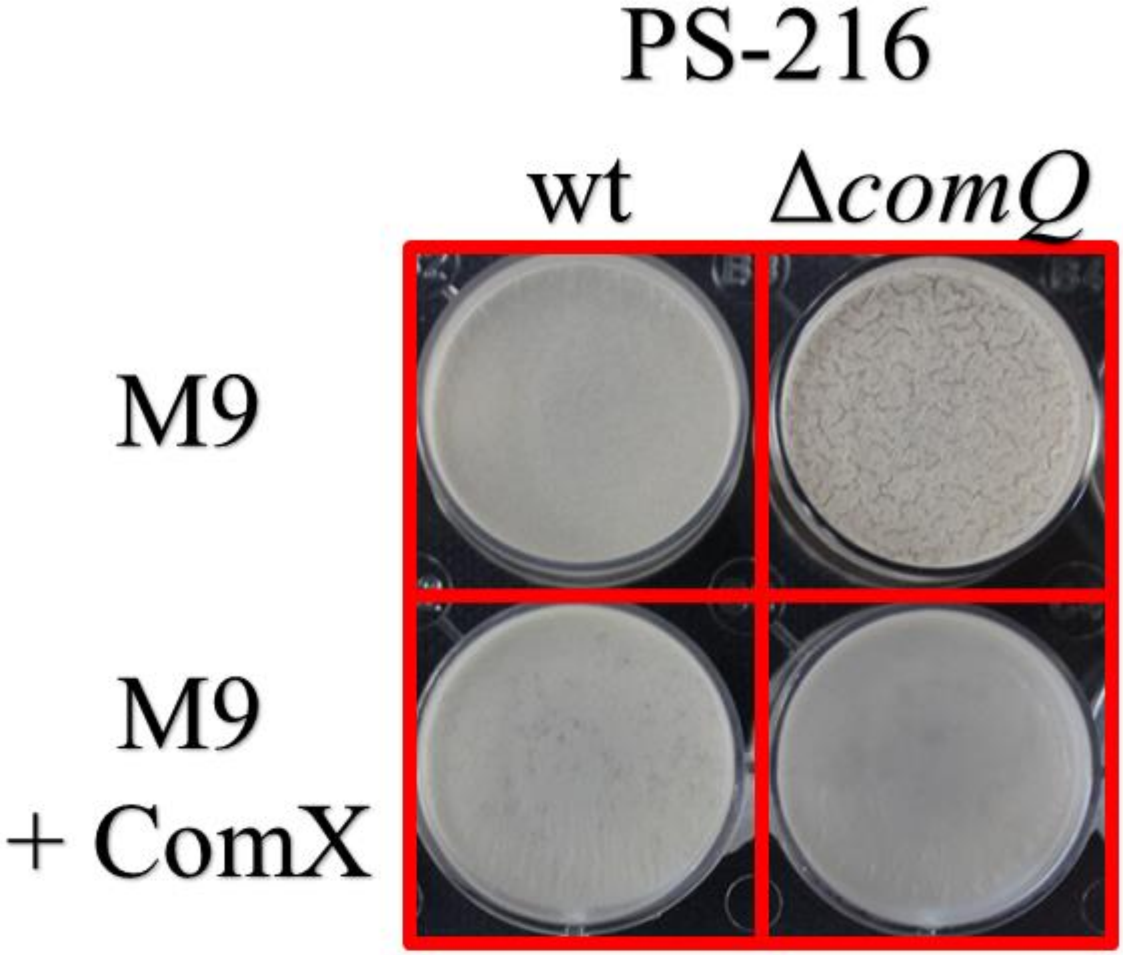

Supplementary Figure 6: Floating biofilms in 12-well microtiter plates grown in MSgg medium complemented with 20% (v/v) spent M9 minimal medium with or without ComX.

Cells were grown in MSgg medium with the addition of M9 *E. coli* ED367 spent medium (20% (v/v)). In the first row, MSgg medium was complemented with spent M9 medium, where ED367 heterologous expression of ComX was not induced with IPTG and therefore the medium lacked ComX. In the second row, MSgg medium was complemented with spent M9 medium, where heterologous expression of ComX in ED367 was induced with IPTG, and therefore contained heterologous ComX. Floating biofilms were grown in 12-well microtiter plates, incubated in static conditions at 37 °C for 24 h.

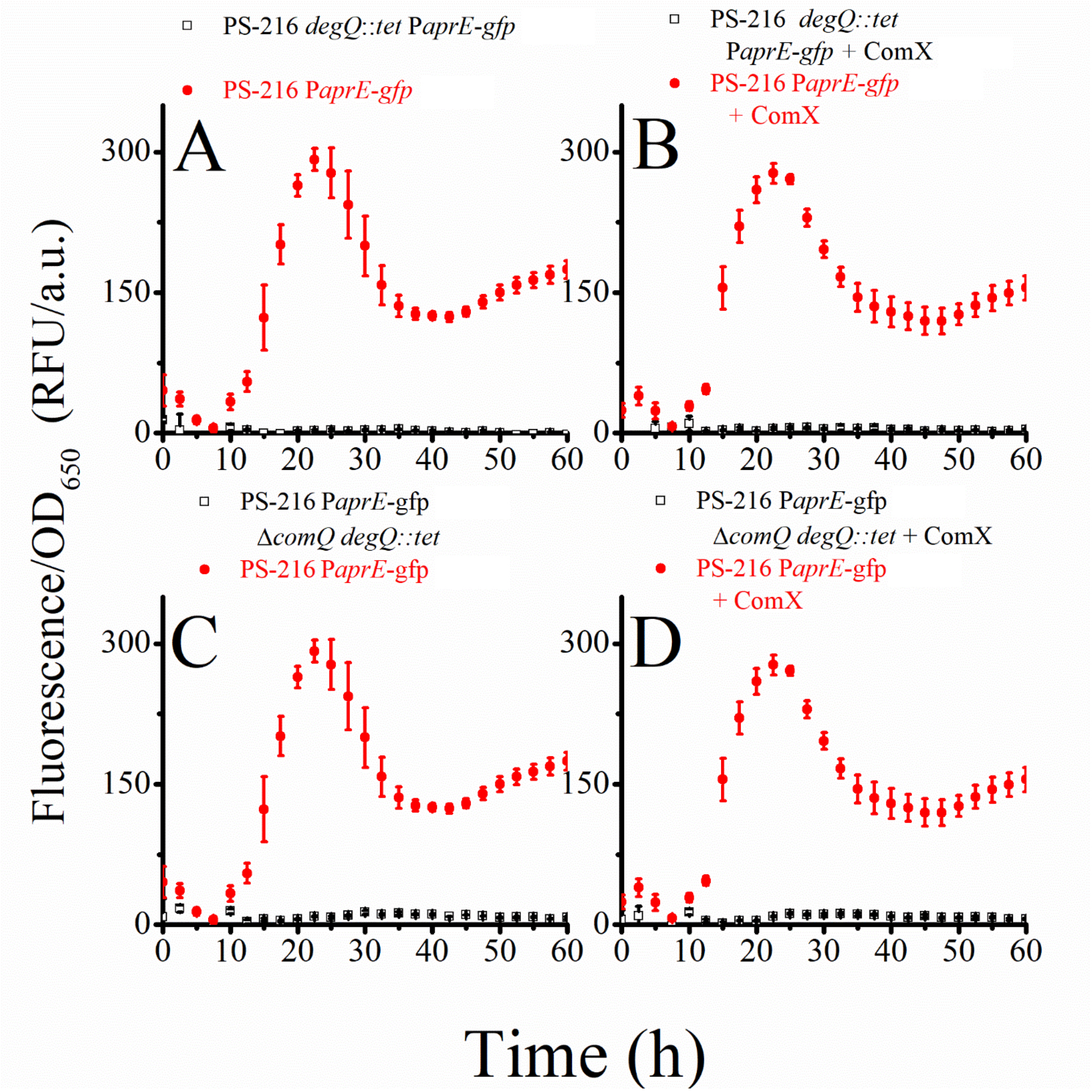

Supplementary Figure 7: P*aprE-gfp* expression of floating biofilms grown in MSgg medium complemented with 20% (v/v) spent M9 minimal medium with (B, D) or without (A, C) ComX.

Cells were grown in MSgg medium with the addition of M9 *E. coli* ED367 spent medium (20% (v/v)). In panels A and C, MSgg was complemented with spent M9 medium where ED367 heterologous expression of ComX was not induced with IPTG and therefore the medium lacked ComX. In panels B and D, MSgg medium was complemented with spent M9 medium, where heterologous expression of ComX in ED367 was induced with IPTG, and therefore contained heterologous ComX. Averages of 3 independent biological replicates with their standard error of means (SEM) are shown. Measurements were made every 30 minutes, but only every fifth data point is shown for clarity.

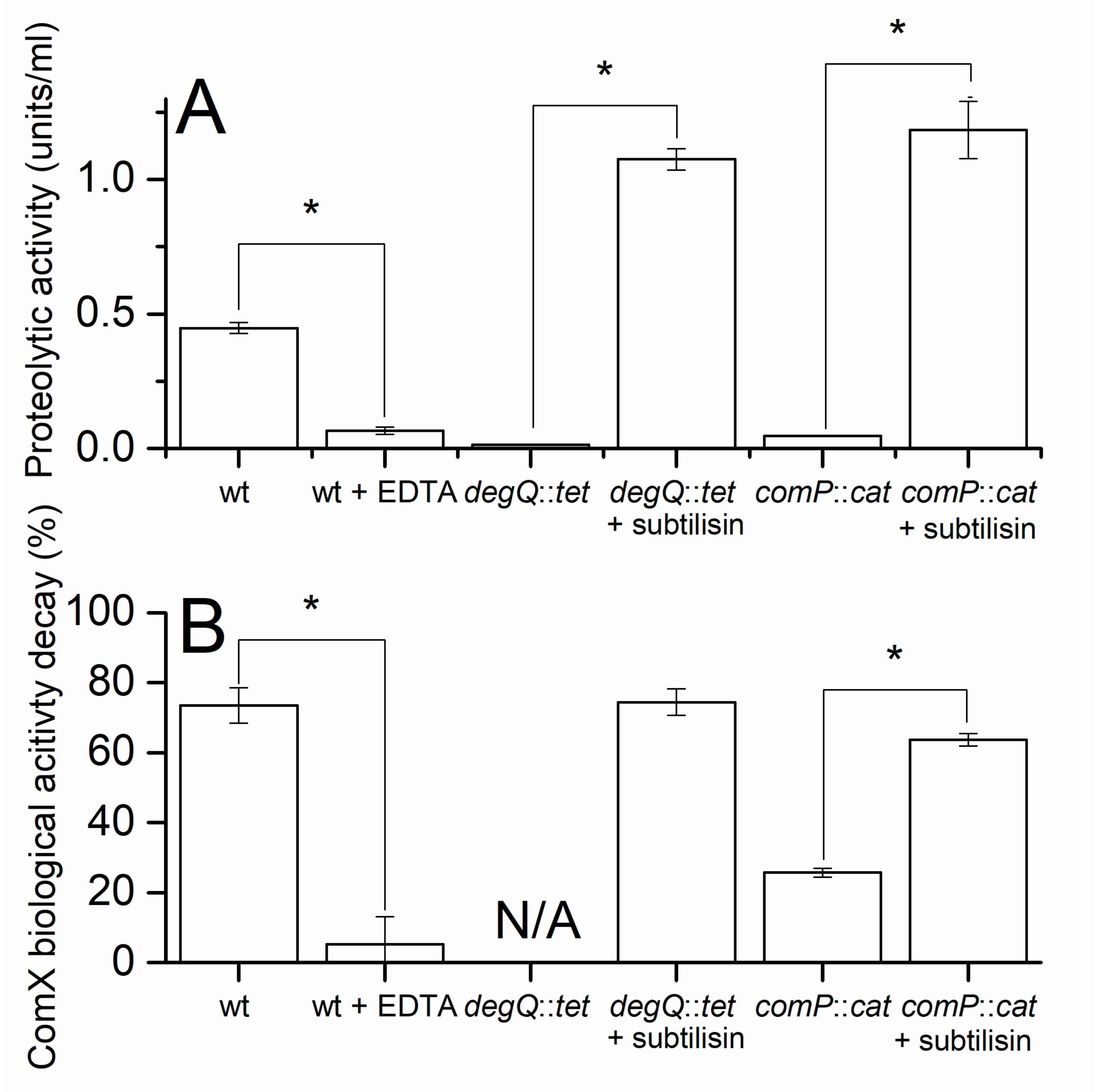

Supplementary Figure 8: Comparisons of the proteolytic activity (A) and ComX biological activity decay (B) of different *B. subtilis* 36 h floating biofilm spent media.

A) Proteolytic activity of the spent medium from floating biofilms. Different *B. subtilis* PS-216 strains (wt, *degQ*:*tet*, *comP*::*cat*) were grown for 36 hours at 37°C in MSgg medium. Where indicated, subtilisin or EDTA was added to spent media after harvest in order to increase or inhibit the proteolytic activity of the media, respectively. Averages and SEM of three independent biological replicates are shown. A Mann-Whitney *U*-test was performed to determine statistical significance of discussed measurements (*p < 0.05).

B) The ComX biological activity decay of the same harvested floating biofilm spent medium as above using a signal deficient biosensor strain BM1456. Averages and SEM of three independent biological

replicates are shown. A Mann-Whitney *U*-test was performed to determine statistical significance of discussed measurements (*$p < 0.05$). N/A indicates that the Com biological activity is below the detection threshold.

1.2 References

Schindelin, J.; Arganda-Carreras, I. and Frise, E. et al. (2012), "Fiji: an open-source platform for biological-image analysis", Nature methods 9(7): 676-682, PMID 22743772, doi:10.1038/nmeth.2019

Schneider, C. A., Rasband, W. S., and Eliceiri, K. W. (2012). NIH Image to ImageJ: 25 years of image analysis. Nature methods, 9(7), 671-675.
